# Adult ADHD in Cultural Ecosocial Niches: Exploring the Rise of Adult ADHD in Context

**DOI:** 10.1007/s11013-025-09958-9

**Published:** 2026-01-19

**Authors:** Jesse N. Ruse, Paul Rhodes

**Affiliations:** Mind and Brain Centre, University of Sydney School of Psychology, 94 Mallett St, Camperdown, NSW 2050 Australia

**Keywords:** ADHD, Ecosocial, Niches

## Abstract

Amid rising adult ADHD diagnoses in recent decades, this article introduces a cultural ecosocial niche theory of adult ADHD, suggesting that symptoms emerge within specific cultural and social contexts rather than solely from neurobiological differences. Through in-depth interviews with seven Australian women recently diagnosed with adult ADHD, complemented by photo-voice methodology, we show how ADHD symptoms fluctuated markedly across different social interactions. The study found that participants actively construct and inhabit cultural ecosocial niches where their traits achieve a functional fit with their social and cultural environment. These niches ranged from adopting the macro-cultural framework of ‘neurodiversity’ to build affirming identities and communities, to finding micro-social occupational niches in fast-paced roles where their cognitive style became an advantage. Each niche was sustained by a reinforcing feedback loop, where the environment (such as a supportive social group or a demanding job) reinforced the very traits and beliefs that initially attracted them to that niche. This study challenges the notion that psychiatric symptoms are confined solely within a person or solely outside in the environment. Instead, it provides a concrete example of how symptoms and their meanings appear at the intersection of both. These findings illuminate the complex ways socio-cultural settings can both constrain and empower, while highlighting implications for how we conceptualise and address adult ADHD in an era of increasing diagnosis.

## Introduction

Attention deficit hyperactivity disorder (ADHD) is a psychiatric neurodevelopmental disorder where individuals display symptoms of impulsivity, hyperactivity, and/or inattention, with the latter being the most common in adults (Wilens et al., 2009). ADHD is primarily diagnosed based on reports of behavioural and cognitive ‘symptoms’ and their effect on meaningful activities (functioning). The most widely used criteria for diagnosing ADHD are found in the Diagnostic and Statistical Manual of Mental Disorders (DSM-5; APA Taskforce, 2013). The DSM-5 states that individuals meet the criteria for diagnosis if they display several symptoms of inattentiveness and hyperactivity, which must have been present since before age 12, and cause impairment in social and occupational functioning.

ADHD was once considered exclusively a childhood disorder; however, it is increasingly diagnosed in adulthood (Fairman et al., 2020). In the early 1990s, ADHD was considered a relatively rare condition in adults, with global epidemiological estimates ranging between 1 and 2% of the adult population (Bellak & Black, 1992). By 2010, these estimates had more than doubled to 2–5% of the adult population (Kooij et al., 2010). In the USA, adult ADHD diagnoses have consistently continued to increase into the present (Chung et al., 2019; Danielson, 2023). This is likely true in Australia, too, where the rates of ADHD medication prescriptions have risen steadily over the past few decades (Bruno et al., 2023). The rate of diagnosis in girls and women has grown at three times the rate of boys and men over the past two decades (Fairman et al., 2020).

A large proportion of children diagnosed with ADHD seemingly ‘grow out’ of the disorder, with up to 80% of childhood cases remitting in adulthood (Sudre et al., 2018). Meanwhile, another group of individuals seemingly develop the disorder (to the diagnostic extent) only later in adulthood. Those with ‘late-onset’ or ‘adult-emergent’ ADHD form a distinct group in the ADHD population; compared to those who were diagnosed in childhood, they are more likely to be female, less likely to have low IQ scores, and less likely to exhibit ‘externalising behaviours’ (Agnew-Blais et al., 2016; Hartung et al., 2022; Moffitt et al., 2015). While the genetic heritability in childhood ADHD is relatively high, for those diagnosed in adulthood, the largest influence appears to be non-genetic (i.e. environmental; Brikell et al., 2015). Some suggest that late-onset (adult) ADHD may be a distinct sub-type of the disorder, where symptoms are clearly observable in adulthood but not salient in childhood (Caye et al., 2016).

### The Problem: Reductionist Conceptualisation of Adult ADHD

Intra-individual theories seek to explain ADHD via neurobiological mechanisms, which are mediated through cognitive capacities, and manifest in behavioural symptoms (e.g. Alexander & Farrelly, 2018). These types of explanations have strongly influenced psychiatric treatments for the disorder, which have focused predominantly on pharmacotherapy and, to a lesser extent, cognitive–behavioural interventions (Weiss & Weiss, 2004). However, despite extensive research, the search for distinctive biological correlates of ADHD is ongoing (Thome et al., 2012). The phenomenon of ADHD is likely to have multiple etiological and neurobiological pathways, and the line between normal and pathological has been described as a “moving target” (Michelini et al., 2022, p. 1). Regardless, even when symptomology is reflected in neurobiology, this is not to say it is caused by neurobiology (Kirmayer & Gold, 2011).

Explanations that narrowly focus on the intra-individual level of analysis offer a relatively thin description of the disorder (Geertz, 2008). These explanations fit a certain technological paradigm in psychiatry, which sees disorder as (i) arising from faulty mechanisms in the individual, (ii) context-independent, and (iii) independent of relationships and values (Bracken et al., 2012). The technological paradigm obscures the role of the social environment, the place where people live, become disordered, and cope. Furthermore, it hides the fact that individuals are socially tethered to others to whom they respond (Di Nicola, 2019). An alternative perspective views cognition as socially ‘situated’ (Smith & Semin, 2004). Mental processes are situated such that they are constantly shaped by the social and environmental contexts to which they are oriented and that provide them with background meaning. A situated account of adult ADHD could highlight the full range of social resources and adaptive strategies to cope with it.

Previous studies, particularly in the medical anthropology literature, have explored the phenomena of Adult ADHD from a cultural perspective (Brinkmann, 2014; Tal & Goodman, 2023) and from a critical perspective (Davies & Horton-Salway, 2016; Hinshaw & Scheffler, 2014; Stenner et al., 2019; Winter et al., 2015). Of particular note is Tal and Goodman’s (2023) study with Israeli adults diagnosed with ADHD later in life. Participants in their study challenged the dominant cultural belief in their context, which frames late diagnosis as problematic. Instead, they framed the time of their diagnosis as beneficial, given that it helped develop various coping strategies, thereby challenging the dominant psychiatric discourse. We wish to explore the cultural and social forces that interact in the Australian context, which might help to understand the recent increase in adult ADHD diagnoses.

### Alternative: ADHD in Context

Material, social, and cultural contexts shape the brains, symptoms, and persons within them (Occhipinti et al., 2024; Patel et al., 2010; Rutter, 1985). Kirmayer and colleagues describe psychiatric disorders as existing in ‘cultural ecosocial niches’ (Gómez-Carrillo & Kirmayer, 2023; Kirmayer, 2005, 2019). The term ‘niche’, borrowed from ecology, refers to the environmental conditions in which a certain species, organism, or behaviour can survive (Chase, 2011). For humans, these conditions are not only material but also cultural and social. A niche occurs when an organism and environment mould each other to fit together in a way that sustains the organism and the environment.

Niche theory suggests that the social environment affects mental processes, while at the same time, these mental processes can bring about change in the social environment. Psychiatric processes (the deleterious mental processes that cause distress and create problems in functioning) are partly caused by an adaptation to a hostile social environment (Patel et al., 2010). However, humans can also re-shape the environment through their actions (Odling-Smee et al., 2013), thereby mediating its effects. Mental processes, including psychiatric ones, are therefore both a cause and effect with respect to what constitutes a niche (Kirmayer, 2015). The process by which niches are constructed is interactive and dynamic, whereby individuals adapt to the social environment, while also altering it to make it more suitable.

ADHD consists of sets of behaviours (symptoms) which allow or interfere with the achievement of some objectives (functioning) which indicate something about the person and the nature of the world (meaning). All these units rely somewhat on processes outside of the brain. Behaviours, such as conversing, problem-solving, organising, and so on, are not enacted alone but are instead mediated through interaction with objects and people in the environment. Whether these actions are ‘functional’ depends on the environment in which they occur. And this behaviour and functioning must be explainable via the stories, symbols, and language that are available in a given culture. Niching, therefore, occurs at various levels, mapping roughly onto the domains of symptoms, functioning, and meaning.

To categorise the contextual forces that affect ADHD, we chose to use a taxonomy of micro- and macro-social levels. This categorisation differentiates between the timescale and social coverage (size) of these contexts. Larger (macro) cultural niches might be available to entire societies and sustain for long periods of time (encapsulated in cultural stories and beliefs). Likewise, an individual might construct a more local (micro) niche that serves their personal needs and exists only within the confines of a single relationship or interaction. Below, we define the meaning of these terms and provide evidence of the effects of these forces on adults with ADHD.

## Two Levels of Context

At the micro-social level are the most immediate interpersonal relationships and material conditions that influence psychiatric symptoms. Here, we refer to the interactions that individuals have with people and objects that mediate the expression of symptoms and provide resources for coping. We largely rely on others to regulate our mood (Marroquín, 2011) and achieve complex tasks (Billings & Moos, 1982). Schilbach (2016) convincingly argues that many, if not all, psychiatric diagnoses involve a failure of reciprocal social feedback loops, wherein symptoms are the result, and cause, of poor social interactions. A lack of interaction with others, or interactions of a problematic type, may result in individuals using other means to address their needs, some of which might be maladaptive or ‘symptomatic’. Evidence from family studies indicates that ADHD is associated with stressful family interactions such as when there is a difficult attachment between parent and child (Erlandsson et al., 2022). Meanwhile, warm ‘authoritative’ parenting protects against ADHD (Modesto-Lowe et al., 2008). Similarly, having a supportive social group appears to buffer against the expression of more severe ADHD symptoms in adulthood (Lan et al., 2023). Meanwhile, adults with ADHD who are situated in workplaces that afford more hands-on and fast-paced tasks can work efficiently and productively, functioning to a high standard (Lasky et al., 2016).

At the macro-social level, large-scale cultural conditions also shape individuals. Macro-social conditions, such as political and economic arrangements, become active social forces when distilled into cultural beliefs and customs (Kirmayer & Bhugra, 2009). Cultural beliefs are the systems of meaning through which the world is conceived and perceived. We focus here on how culture provides causal explanations of behaviour and pathology. The availability of meanings for psychiatric phenomena changes relative to moral and ethical dimensions (e.g. the pathologisation of homosexuality; Bayer, 1987), political situations (e.g. drapetomania; Szasz, 1971), and financial incentives (e.g. the chemical imbalance theory of depression; Lacasse & Leo, 2015).

With regard to adults with ADHD, the diagnosis has been associated with low-income groups in precarious living situations (De Zwaan et al., 2012; Spencer et al., 2022). Cross-culturally, however, adult ADHD is less prevalent in low-income countries than in high-income countries (Fayyad et al., 2007).^1^ One interpretation of this finding is that certain diagnoses arise in response to the types of healthcare that are available to treat them (Conrad & Bergey, 2014). With regard to gender arrangements, adult women represent the fastest-growing group of ADHD diagnoses in Western countries (Simon et al., 2009). One interpretation of this finding suggests that women who are exposed to the gendered expectation to balance work, household, and family demands (the impossible ‘superwoman ideal’) can experience a splintering of attention and difficulty functioning relative to these high expectations (Winter et al., 2015).

## Research Aims

This study aims to provide empirical evidence for the cultural ecosocial niche perspective concerning adult ADHD. We aim to highlight instances of niching that influence the symptoms, functioning, and understanding of adults with ADHD. To do this, we aim to map the cultural ecosocial niches where ADHD occurs, showing how cultural and social contexts contribute to and alleviate the disorder. A secondary aim was to highlight how this perspective might enable individuals to access emancipatory resources to alleviate distress associated with ADHD. We aim to highlight the niche construction process: the ways that the environment is manipulated to be more enabling to those living with ADHD.

## Methods

### Participants

The participants were seven adult Australian women who had been diagnosed with ADHD in the previous 12 months. Participants self-reported their ADHD diagnosis and any co-morbid psychiatric diagnoses they had been given. Participants were recruited via a Facebook post on the group ‘Adult ADHD Australia’. The advertisement asked for anyone over the age of 18 who would like to participate in a study exploring how context affects ADHD. A total of 20 people replied to the advertisement, ten of whom returned the participant questionnaire that was attached to the information and consent form. Of those, seven made a time for the initial interview. One participant did not return for the second interview. The internal ethics committee of the host university approved the study (HREC 2023/388).

This sample, recruited via convenient means, was demographically characteristic of the rise in new adult ADHD diagnoses in Australia (see introduction). All participants were female, though this was not a requirement for participation. All participants were working in professional jobs, though one was semi-retired. Information regarding participants’ socio-economic status was not recorded, though all appeared middle-class, as was partially indicated by their ability to obtain a diagnosis for ADHD (in Australia, where individuals self-fund their assessment). Moreover, all women appeared to have high ‘health literacy’ (Berkman et al., 2010), again, partially indicated by their ability to navigate the health system and seek a diagnosis. The Facebook-recruited sample exhibited comfort with social media, possibly indicating a preference for this medium as a means of connection and communication. Some participants identified as being in relationships, some with children, while others did not. These demographic details contextualise the findings. Participant characteristics can be seen in Table 1.

**Table 1:** Characteristics of study participants

Participant	Age	Occupation	Date diagnosed	Medication?	Any mental health diagnoses
1	44	Rescue operations coordinator	Jun-22	Lisdexamfetamine	No
2	42	Kitchenhand	Jul-23	No	DAS*
3	33	Management consultant	Nov-22	Dexamphetamine	No
4	30	Bush regeneration	Feb-24	Dexamphetamine	No
5	58	Copywriting consultant	Mar-23	No	No
6	26	Business development coordinator	Mar-23	Lisdexamfetamine	DAS*
7	27	Student/caseworker	Nov-22	No	OCPD**

*Depression and anxiety, **Obsessive compulsive personality disorder

Participants were interviewed twice by a member of the research team (first author), both times via online video call. The first interview was semi-structured. Participants were asked about their lives outside of ADHD (including their work, relationships, and living situation), about the period preceding their diagnosis, and about their life following diagnosis. Particular attention was paid to how ADHD fit into the wider context of their lives, both before it was labelled as such and after understanding it as such. Participants were then asked to keep a photo diary for the next four weeks, taking photos of situations where they felt ADHD showed up for them and times when their lives felt unaffected by ADHD. This prompt was deliberately ambiguous, to allow participants’ own conceptualisation of what terms like ‘ADHD’ and ‘show-up’ mean to them. The second interview involved reviewing these photos together. Most participants had fewer than a dozen photos from the month, although one had over 50. The interviewer prompted the interviewee to explain each photo to them, dialogically exploring the context of the situation together. The interviewer’s prompts were open-ended, such as “tell me what’s going on here” and “Can you explain this photo to me?”, wherein the interviewee would then tie the photo back to ADHD in a personally meaningful way.

This established method, sometimes called photo-voice, has been used to deepen and extend participants’ narratives by providing reminders of their experiences that can be explored in interviews (Han & Oliffe, 2016; Thompson et al., 2008). This is perhaps particularly important for those with ADHD who are able to overcome memory deficits when aided by visual prompts (Skodzik et al., 2017).

Moreover, as Thompson et al. (2008) suggest, “photo-voice enlarges the perimeters of health from the strictly medical to the psychological, economic and social conditions” (p. 15). This capacity makes the methodology particularly well suited to an ecosocial approach. By using photos as prompts, the interviewer could draw participants’ attention to the specific elements of their environment. The explicit focus became the people, places, and things that participants interacted with and how this connects to their inner experience. This process allowed for the exploration of multiple lived contexts of participants’ lives over a one-month period. This provided a richer and more ecologically grounded exploration than would have been achievable by interview alone.

### Interpretative Analysis

Qualitative data were analysed to examine the fit between the individual and the social, material, and cultural environments. The fit was examined by exploring the interactions between the individual and the environment as they manifest in participants’ behaviours, emotions, and cognitions. In fitting with the ecological perspective, these interactions were considered to be bi-directional. Participants’ emotions, for example, were conceptualised as being caused by contextual factors while simultaneously causing changes in the contextual environment (Krueger, 2014). Cultural beliefs were conceptualised as forces that affect participants’ interpretation of the world but were also considered as ‘resources’ that could be used to justify or negate some course of action (Laland & O’Brien, 2011). Rather than being conceived of as internal states, participants’ thoughts and feelings were viewed as emergent properties of the ongoing process of niching—active processes that are adaptations to, and shaping of, the environment.

With this perspective in mind, analysis of the interviews followed a reflexive thematic analysis approach (Braun & Clarke, 2006). Transcripts were analysed on a line-by-line basis, coded regarding smaller units of meaning, and these codes were then sorted into emerging themes concerning the interaction between the person and the environment. This process was iterative, resulting in further themes being added and transcript segments re-sorted with respect to these new themes. The analysis was reflexive, such that the themes were generated from within a certain perspective, one that considered psychopathology to be connected to social and cultural forces.

## Findings

Participants described pockets of goodness-of-fit (ecosocial niches) where everyday activities, such as socialising online, producing creative work, or unwinding, were made easier in certain contexts. These ranged from short-term micro-contexts, such as contemporary media ecologies, to more enduring macro-contexts, such as fast-paced career roles. These accounts of situated ‘success’ were produced by a match between attentional style and environmental structure, not as evidence of colloquial ‘individual success’. Importantly, participants also described misfits (e.g. organising meet-ups, sustained messaging), underscoring that fit is often partial and domain-specific.

### Macro-level Cultural Contexts

At this broadest level, participants’ experiences were shaped by cultural meanings concerning identity, causation, and diversity. These frameworks provided meanings for ADHD to become intelligible and were used to actively construct social identities.

### The Niche of the Diagnosed Self: Fitting Biology to Biography

Participants consistently framed their actions and identities as partially determined by brain chemistry. They frequently attributed both positive and negative behaviours to their dopamine levels or neural wiring. Brain chemistry was overwhelmingly cited as the fundamental cause of ADHD. This biological framing positioned certain tendencies as somewhat beyond conscious control. This provided justification for departures from dominant cultural norms, such as remaining quiet in meetings or showing deference in social hierarchies.

I try to explain it that my brain is just seeking dopamine. So I like to do things that are interesting and exciting. So if I’m doing a job, I’m now looking for a job where I’m not going to be doing repetitive stuff, stuck at a desk. And, sorry, sometimes the filter goes off in social situations. So if I say something that you find offensive, just let me know. And I’m comfortable saying that. And that I’m gonna forget stuff. So, you know, it’s not that I don’t care, I’m just going to forget things. So apologies. I’m doing my best but I’m just... these things aren’t going to happen [Participant 2]

Within the broader category of brain-based explanations, participants frequently used psychiatric diagnostic frameworks to explain and justify life trajectories. This was particularly the case when they diverged from conventional expectations. One participant attributed her exploration of multiple university degrees and professions in her twenties to ADHD, noting this differed from the expected path of early professional specialisation and linear career advancement. Participants would suggest that their life trajectories, particularly when they deviated from unrealistic career and relationship expectations, must be viewed through the lens of psychiatric diagnoses. Psychiatric language was used to deviate from normative expectations without directly challenging those norms.

This diagnostic framing created a niche that provided relief from certain expectations while avoiding direct criticism of social pressures. For example, when asked about contextual factors influencing her life, one participant positioned her diagnoses as mediating influences:

[Interviewer]: You said that you believe that when people grow up and go through adolescence, society and culture start to have a bigger impact on people. I think you said society can be “oppressive” sometimes? Did you feel that that was the case for yourself growing up?

[Participant 2]: Yeah, I think so. I have always put a lot of pressure on myself. And I think that comes back to my diagnoses [OCPD and ADHD]. There’s this expectation of being perfect. Doing well in school, having a good job, having good friends, acting a certain way, being a certain way. And when you’re not able to do that, well, then that makes you weird. You’re different. You’re an outsider.

A core reinforcing loop was observed between participants’ belief in a brain-based personhood and their use of diagnostic causal attributions to narrate their biography. These two cultural meanings were deeply intertwined and strengthened each other. The belief that identity is rooted in biology and the practice of using a diagnosis to explain one’s life story thus exists in a feedback loop, creating a stable social niche. The psychiatric–determinism framework provides the tool for justification, and each time that tool is used successfully to make sense of one’s biography, it validates and solidifies the initial belief that the self is fundamentally biological.

### The Niche of Neuro-social Belonging: Fitting Identity to Community

While participants often used diagnostic language, they also showed a strong affinity for the emerging “neurodiversity” discourse, particularly when constructing their social identities. They frequently spoke of being neurodivergent rather than having ADHD. Participants’ embrace of the neurodiversity discourse allowed them to construct an affirming identity niche where ADHD is positioned as a natural biological variation rather than an inherent problem. They emphasised the “natural” diversity of neurotypes and advocated for acceptance of non-dominant cognitive profiles. Unlike the psychiatric discourse, participants positioned neurotypes as immutable aspects of identity, a framing which supported advocacy for social-political acceptance rather than personal change. Attempts to conceal one’s neurotype (described as “masking”) were framed as potentially psychologically harmful. For example, participants often rejected pathologising psychiatric language when asked about ADHD “symptoms”.

It’s not symptoms of a disorder, it’s just traits of a neurotype. That just means it’s the kind of brain that I have. Because I’ve never felt like I have a disorder. It’s a neurobiological condition, is what I understand now. But that just means it’s the kind of brain that I have. And I hate [the term] “symptoms”. I say “traits”. So, things that make life difficult for me and things that make life easy for me, just like any other brain. But you can group certain types of brains into different areas, and ADHD is one of those groupings. So yeah, I don’t see ‘symptoms’. I see ‘things that work for me, things that don’t work for me.’ Things I do well, things I can’t do well. Just like a normal person. [Participant 5]

This identity provided a powerful rationale for reshaping their social worlds. Participants sought meaningful belonging by forming connections with other people they identified as “neurodivergent”, a process which might be called neuro-social grouping. This practice of curating their social circles based on a shared neuro-identity was complemented by neuro-social distancing, where the “neurotypical” category was used to understand difficult relationships. By linking relationships through the shared sign of “neurodivergent”, participants would describe shared eclectic behaviours or interests as to why some social arrangement (with a friend, or spouse, or group) worked well. The identity provides the logic for these social actions, and in turn, the social validation received within these like-minded groups powerfully affirms and strengthens the neurodivergent identity, making it a more robust part of their self-concept.

So I went to visit some friends on their farm on the weekend, and that was just delightful. [Pointing to the picture] This was someone I went to school with, who, I can see now, definitely has ADHD. When she actually heard about it, she’s like ‘yep!’. Her daughter’s been diagnosed, and I was like, ‘Oh, I wonder where she gets that from.’ So, we’ve been really good friends for a very long time and hadn’t seen them in years. We went to this farm with them and the conversation just flowed. We laughed a lot. It was just fantastic. So I guess that’s just a picture of neurodivergent people getting along well [...] I’ve never had trouble finding neurodivergent people to become friends with. Most of my friends, I think, they either know or they don’t know [they are neurodivergent]. But I think most of them are neurodivergent spirits. Certainly the ones I’m closest to. In the process of my own self-diagnosis, I worked out that my husband is autistic, which he has confirmed. As we have grown to learn more and more about neuro-divergence he has confirmed that ‘yeah, that that does sound right.’ [Participant 5]

The adoption of a neurodivergent identity and the practice of neuro-social grouping were observed in a synergistic and mutually reinforcing relationship. Participants’ adoption of this identity justified the social action of grouping themselves with neurodivergent others and distancing themselves from neurotypical others. It gave participants a clear rationale for why they might feel more comfortable with certain people rather than others. In turn, that social action (grouping with those they like) gives substance to the identity construction, making it more real and meaningful.

### Micro-social level

At the most proximate social level, symptoms and behaviours were sustained by social niches where personal history, adaptive behaviours, and environmental features fit together.

### The Niche of Self-sufficiency: An Adaptive Fit to Social Isolation

Participants reported a history of significant challenges in fitting into social collectives, particularly those with gendered expectations for agreeableness and politeness. Instead of conforming, participants often challenged social hierarchies and norms, which appeared to produce a sense of social isolation. This was evident, for example, when one participant reported a fundamental lack of “relatability” with peers, while another participant attributed this to missing the “social rule book”. Notably, participants typically attributed their isolation to personal deficiencies rather than social dynamics, citing tendencies to, e.g. “overthink” interactions or be “over-empathetic”.

I think having ADHD, I always just miss out. So there’s always something that I’m blaming ADHD because I’m not a ‘yes person.’ I have a real sense of justice. So if something’s not right, I’ll speak up. I call people out on stuff. That’s seen as being difficult. When actually I’m not, I’m just trying to share knowledge. And it doesn’t have to be something that impacts me, it could be something that impacts someone else [...] so I don’t have a lot of female friends I’ve always gotten along better with guys. I do have some close female friends but I don’t have a group of girls, that like, go drinking. You know how people have a girl gang, I don’t have that. And the females that I am friends with a little bit different. [Participant 6]

Like, as a young person, I didn’t have many friends. I didn’t quite know how to make friends. I didn’t know how to talk to people. Because small talk is just not my jam. Do you just go up and say hello to someone? What if they don’t want you to say hello to them? [...] It’s hard not having any friends as a kid and wondering why people won’t talk to you. I didn’t like sitting alone. Because I felt like everyone was staring at me because I was sitting alone, which made me more of an outcast [Participant 2]

On the other hand, participants developed a robust capacity for self-sufficiency, often reframing their independence as a strength. They gravitated towards groups that valued self-sufficiency and practical competence over social conformity. Often, they reported developing robust capacities for self-entertainment and self-soothing. Their favoured activities often involved a lot of autonomy, such as woodworking, crafts, independent research, solo physical exercise, musical instrument learning, and entrepreneurship.

I’m a volunteer firefighter. Have been for 10 years now. I’ve been to some big bushfires up in Sydney. Being a female in a very male-dominated environment... Like, I turn up, I had pink hair and sparkly long sparkling acrylics on, they’re like ‘What the hell’s this?’ I didn’t do as much as the boys. That course I was on there [showing me a picture of a fire-training course], I was the only female. I actually lead one of the captains of the other brigade through the assessment, to get through the little house on our hands and knees. That morning there, they made us climb a big flight of stairs to get our physical activity up. They made me do that and I freaked out. I wanted to take the [oxygen] mask off. I had to say to myself ‘You’ve got this. You’d look like a dickhead If you take it off. You’re the only female here, you’ve got to do it. Do it for yourself. But you really need to do it for the other girls.’ [Participant 1].

Participants’ experiences of social isolation and their highly developed sense of self-sufficiency were intertwined in a self-perpetuating cycle. These two phenomena mutually reinforced each other to create a self-sustaining niche. Difficulties in social engagement make a self-sufficient lifestyle both necessary and attractive. In turn, a life successfully built around autonomous work and solo hobbies can reduce the opportunities and motivation for the types of broad social engagement participants found challenging, thereby reinforcing their independence and social distance.

### The Occupational Niche: Fitting the Environment to Attentional Needs

Participants’ ADHD symptoms often intensified when they attempted to complete complex tasks without interpersonal resources. One participant described feeling overwhelmed when inheriting her late mother’s belongings and trying to manage the sorting and selling process solo, while another described the emotionally charged task of taking her children to swim school with little support. This dynamic was especially clear when salient social cues were removed, as when participants were forced to work from home. Without the conspicuous social scaffolds of the office, they struggled to maintain focus.

I was working at home on a Thursday and at 10am during my workday, I said ‘I’m gonna cook a lentil dal.’ And so this [showing me a picture of the dal] represents my inability to concentrate. So yeah, here I am. I’ve got so much to do at work. But I decided to take an hour out of my workday to cook a lentil dal that I was going to eat later on [...] so I literally had to ban myself from working from home because I’m so unproductive. Because I just want to do other things. Like whatever is interesting to me at the moment in time. No one’s there to hold you accountable. So, I still allow myself to work from home, but I make it a priority to go into the office. So that I don’t have the temptation to do something else. But it still that happens. But, yeah, like you’re kind of reminded that you’re at work and you’re like ‘ok, pull it back’ [Participant 6].

The relationship between the presence of external scaffolding and participants’ functioning operated in a clear feedback system, defining the boundaries of their effective niches. The success experienced in structured environments and the failure experienced in unstructured ones mutually reinforce the understanding that such scaffolding is essential. This understanding then guides future choices, leading participants to actively seek out scaffolded environments. Conversely, symptoms often diminished significantly when participants surrounded themselves with others. Several described improved learning and consistency in the presence of others, for example, mastering an online course in a group setting or maintaining a Pilates practice by attending classes rather than practising alone.

### Functional Interaction Niches: The Fit Between Cognitive Style and High-paced Environments

Participants’ ability to “think so quickly, and react really quickly” made them a good fit for unpredictable, intense occupations. Their work was typically hands-on and fast-paced; among those in the study were a gymnastics coach, bush regenerator, call centre operator, child-protection officer, kitchenhand, disability support coordinator, and emergency services coordinator.

Okay, this is a photo of my cousin. And he has a very rare disability. And the reason I’ve put this [photo] in is he has very high needs and is very unpredictable. And I think that I’m quite suited to something like this. You know, this high-paced environment, because I have the ability to think so quickly, and react really quickly as well. Like, if he falls, and you know, he’s bleeding or you know, he’s having a seizure, I think that in really high-stress situations I have the ability to be calm. I think, day-to-day, my brain is moving so fast. And when something crazy is happening, it’s almost as if, like, I am calm. [Participant 6]

This cognitive style, suited for rapid and responsive work, is the same style that aligns with the structure of hyperlinked digital environments. Thriving in a fast-paced job sharpens a certain attentional regime which uses short bursts of attention and frequent task-switching. This is perhaps what made interactions on digital media feel natural and manageable. Most participants discussed using social media platforms like Instagram and TikTok for work, socialising, and entertainment. Several described using social media entrepreneurially, managing family business pages, promoting professional services, or running promotional accounts for workplaces. This creates two distinct but mutually reinforcing functional niches, where an attentionally agile cognitive profile enhances the capacity to thrive in quick interactions, such as on social media.

[participant 2 telling a story about running the social media account of a small business]: I guess it started off as a heated conversation with [business owner], because he was possibly insinuating that he wanted more social media. And, because I’ve done my research, I was able to articulate like, no, it’s actually, you need to be consistent. But you know, there’s no point posting on Monday because no one uses Instagram on Monday. As per the study, I’d read and yeah, that was quite good that actually done my research and the, I guess the hyperfocus had served me well. [Participant 2] A niche was observed between participants’ perceived fit in fast-paced occupations and their patterns of engagement with digital media, with each context rewarding short-burst attention and rapid task-switching in ways participants felt suited their attentional style. Notably, digital media also produced frustrations (for example, difficulties convening meet-ups or sustaining dating-app exchanges), which some participants themselves attributed to ADHD.

## Discussion

Following (Kirmayer, 2024), we conceived of diagnoses as existing at the intersection of multiple social and cultural forces, deemed cultural ecosocial niches. The findings highlighted relevant factors at each level of analysis. In their micro-social environment, participants tended to miss out on rich social interactions through misstatement or exclusion. They therefore attempted to do complex tasks without help, leading to disorganisation and procrastination. Yet, with the aid of others, in the form of highly variable social interactions or digitally mediated platforms, their attention was guided back to relevant activities. Participants were often able to find and create occupational environments that were rich in just these types of tasks. Yet, their way of relating to others stood in contrast to social norms, often of a gendered nature, which demanded a kind of etiquette and comportment that they eschewed. Despite feeling different from their peers, they adapted to this by pursuing independent activities, hyper-focussing on solo tasks, and finding refuge in groups that prized self-sufficiency. At the macro-social level, there was a niche for this way of being in a culture that allows for ‘difference’ based on biological and neurological grounds. Hence, participants found identity and inclusion through the neuro-diagnostic paradigm.

Participants were situated within a national context where diagnostic labels were pre-legitimised by the mental health industry, which has reasonable standing in Australian society (Brauer et al., 2021). Progressive health media campaigns in Australia have aimed to destigmatise mental health conditions (Hickie, 2004). There has been a particular emphasis on identifying and intervening for those who are ‘at risk’ of disorder, even if they do not meet full criteria for a diagnosis (McGorry et al., 2014). Emily Martin (2007, Chapter 8), in her analysis of depression and mania, suggests that diagnostic labels can shrink and expand, particularly when they are tied to economic outcomes, thereby allowing more or fewer people into the diagnostic category. In Australia, recent expansion of most psychiatric labels, including ADHD, has effectively allowed for a larger portion of the population to engage with the psychiatry industry.^2^ It was not surprising, therefore, that participants often conceptualised their suffering as a form of psychiatric disorder and used diagnostic labels endorsed by authoritative psychiatric institutions.

In particular, participants often had a form of self-understanding epitomised by belief in the “neurochemical self” (Rose, 2003). Rose argues that cultural and economic forces have promoted an agenda that locates psychopathology within the brain. Indeed, popular representations of ADHD often characterise the disorder as a “problem of the brain” (Horton-Salway & Davies, 2018). Following this, individuals re-code their own behaviours as being neurochemically caused (see also Vidal & Ortega, 2020). Brinkmann (2016) describes a cultural phenomenon of “psychiatrisation from the ground up”, whereby individuals apply diagnostic labels to themselves and readily urge others, including psychiatrists and psychologists, to do the same. This cultural niche, where psychiatric labels are pre-legitimised and self-adopted, sustained the cultural meaning of ADHD as something biologically inherent and personally meaningful.

A psychiatric diagnosis, and a neurochemical one in particular, could sustain because it provided some refuge to those who felt “separate from mainstream society” (as one participant put it). Participants described experiencing confusing social interactions and, at times, blatant ostracization. Participants could justify their actions by their diagnosis and thereby gain some allowance to follow a non-dominant path through life. This was particularly true in a society such as Australia, where there has been a continuous push towards neoliberal social policy that values ‘blind’ meritocracy (Western et al., 2007). In such a culture, there are few remaining ways to validly justify non-achievement in education, career, and social life. Most described feeling greatly relieved to get a diagnosis of ADHD. Participants found a niche within a culture where psychiatric diagnoses are legitimate reasons for alternative ways of achieving.

This kind of understanding, however, also divorced participants from their social environment. When human actions appeared neurochemically caused, the emphasis on change lay within the brain, not outside in the social environment. This was highlighted by another finding in the study: participants’ symptoms were sometimes the result of trying to do complex tasks without help from others. By understanding their own behaviour through a neurochemical or neurophysiological perspective, the socially contingent variation of their symptoms was obscured from view. Internal shortcomings, such as “dopamine seeking” tendencies, were often blamed for challenging experiences (like stressful outings with children, struggles in adapting to new work methods, or failing to complete college degrees). Yet many of these tasks required the cooperation or emotionally soothing interpersonal influence of others.

Grange Isaacson (2020) describes a mixture of cultural and social forces that obligate ‘hyper-independence’, using the paradigmatic example of her native Usonian context. She describes problems that develop when people can’t rely on others, resulting in the development of pathological tendencies to cope with forced independence (see also Peacock et al., 2014). A mix of social preference for independence, plus a lack of interpersonal resources, led some participants to struggle to complete actions that, in different circumstances, might’ve been completed cooperatively with others.

The findings from this study suggest that there is also a comfortable and functional niche for such independence. Not only did diagnosis provide a coherent cultural logic for their suffering, but it validated a different way of being. Participants developed ways to entertain themselves, learn by themselves, and soothe themselves. In their relationships, they could eschew implicit demands for harmonious and implicit communication and instead be brazen and explicit about their views. They pursued solo hobbies, such as woodworking, sewing, and martial arts. In their jobs, they reported doing comparatively better in areas where they could make use of their ‘hyperfocus’; the ability to ignore social interferences and deep-dive into solo work. By creating a justifiable reason for separation from others, participants were vindicated in their preference to be self-sufficient and shamelessly individualistic.

Participants often reported ‘feeling different’, which is consistently reported in adults with ADHD across the lifespan (Brod et al., 2012; Ginapp et al., 2023; Nyström et al., 2020). The findings from this study are consistent with research that suggests that adult ADHD and loneliness are connected (Stickley et al., 2017). Participants described feeling unaccepted within peer groups and excluded from shared activities. They somewhat attributed this to quasi-psychological social deficits (e.g. being an overthinker, over-sensitive, lacking ‘communication skills’). Finding inclusion was perhaps particularly important to those in this study who appeared to experience subtle social rejection, particularly in their adolescent years. This niche of independence, perhaps promoted by cultural normative ideals, was challenged somewhat by the neurodiversity paradigm.

Despite the discursive transformation brought about by the destigmatisation movement (Angermeyer et al., 2017), a completely psychiatrized identity was neither affirming nor emancipating. The metaphor of mental *illness* is still rooted in a disease paradigm. The ‘deficit model’ of mental illness culturally denies the possibility of celebrating such conditions. Moreover, when one’s actions are seen as symptoms of potential sickness, the afflicted individual is potentially sapped of political power. Instead, the neurodiversity paradigm uses the ecological metaphor of biodiversity to describe ADHD. ‘Biodiversity’ was a term used to advocate for the conservation of all species, following from the understanding that variability in living things is desirable for a thriving ecosystem.

The concept of ‘neurodiversity’ has biological undertones and fits well within a society that values secular and scientific belief systems. It also fits alongside other modern Western cultural movements that promote acceptance over assimilation, such as multiculturalism and LGBTQIA+ rights movements. Being a particular ‘neurotype’ meant having a hard-wired, natural, and informative identity. The neurodiversity model shifted the focus from trying to find a ‘cure’ to helping facilitate social- and self-acceptance. Participants could understand their place in the world via a belief system that didn’t relegate their status to something deficient. Participants’ struggles resulted from being ‘out of place’, rather than ‘broken’. Effectively, this created the conditions in which participants could find a positive identity and could be accepted and appreciated.

The social category of ‘neurodivergent’ allowed for the creation of a social identity within an ‘in-group’ (Tajfel & Turner, 1986). Having an ‘in-group’ made it possible to find solidarity by drawing attention to the similarities, and away from the differences, between participants and their friends. Similarly, it helped them reconcile differences between themselves and ‘neurotypical’ others. By neuro-categorising others, participants modulated the social environment by pulling some people in closer while pushing others (e.g. ‘neurotypicals’) away. The neurodiversity paradigm also provided for the community through its symbolic power to connect. When participants perceived a relationship as being between neurodivergent people, they could enact their preferred social style with less hesitation and assume a greater level of familiarity with their interlocutor. It imbued certain social interactions with extra meaning and thereby made these relationships stronger. Participants were able to find, and create, a social niche with others whom they felt understood by and comfortable with.

Online social media was one way that social interaction was preferable for those in this study. Digital media use (in particular, on smartphones) appears to be connected to self-reported ADHD symptoms (Kushlev et al., 2016; Ra et al., 2018). Internet use is associated with a type of attentional regime that favours short attentional bursts with frequent switching between online media content (Firth et al., 2019). This differs significantly from pre-digital written and oral traditions, where a premium is placed on sustained attention on a single medium. Given the study’s rich descriptions of digital engagement and the salience of certain identity discourses, recruitment through a social media platform may have shaped both who participated and the kinds of discourses and resources available to them. Because the sample was recruited via Facebook, and because social media affords both identity resources and attention-friendly formats, participants may have been especially likely to encounter fits in these environments. We therefore treat these accounts as contextual and transferable with caution, not universal.

The functional use of modern media technology can represent an adaptation to an environment that places a premium on attentional agility. Australian psychiatrist Gordon Parker has suggested that “digital pre-occupation and increasing pressures for a 24/7 online presence may be setting a variant trap and springing a new variant of ADD/ADHD [adult onset ADHD] into existence” (2024, p. 9). The findings from this analysis are somewhat less alarming. While it did appear that participants engaged with digital media, it is unclear whether this was unambiguously dysfunctional. They used social media technologies to socialise, dating apps for relationships, internet sources to research, and gaming platforms for leisure. Meanwhile, digital technology conditioned attention to be agile, so that it could be functional for continued technology use. Agility in attention could either be functional or dysfunctional depending on the demands of the environment.

Participants found their cognitive profile allowed them to excel in work that was high-pressured and unpredictable. They were able to split their time over several different tasks, such as when they switched back and forth between jobs or between paid and unpaid labour. These findings are consistent with (Lasky, 2015; Lasky et al., 2016) who found that adults with ADHD could engage their symptoms in positive ways when they engaged in work that was challenging, required multi-tasking, and was fast-paced.

## Summary and Conclusion

The cultural ecosocial niche perspective is an emerging theory being used to understand psychiatric phenomena (Gómez-Carrillo & Kirmayer, 2023). This study contributes to the ecosocial niche literature by highlighting concrete examples of niche construction, using the case of ADHD. Moreover, this study showed *how* these niches become stable and self-perpetuating, thereby explaining the continuity of psychiatric disorders over time and place.

In this discussion, we explored evidence for a holistic, ecological model of ADHD, guided by the understanding that cultural and social forces create constraints and opportunities. This model highlights how constraints at one analytic level (e.g. macro-social level) modify other contexts (e.g. interpersonal interactions), leading to re-enforcing feedback loops in multiple directions. For example, cultural beliefs about neurochemical identities modulated the social environment (via neuro-connecting and -distancing) where participants emphasised the similarities between themselves and their neurodivergent interlocutor, thereby re-enforcing the validity of these cultural categories. Similarly, feedback loops can begin at the micro-social level. For example, participants had histories of difficult social interactions, which led to engaging in jobs that required independence and autonomy, which contributes to cultures of individualism and self-sufficiency, which leads to individuals missing out on rich social interactions.

The findings from this study challenge the perspective that ADHD develops from the ‘inside-out’—where naturalistic internal causes indeterminately produce behavioural symptoms, leading inevitably to outside (dys)functioning, which is then interpreted via cultural meanings. Instead, an ecosocial theory conceptualises health condition (such as ADHD) as an emergent property of multiple, overlapping contexts. Not only does it eschew a reductionist account of ADHD as being merely an internally caused (i.e. neurobiological) condition, but it also rejects the possibility of reducing it to merely external factors (e.g. merely a social/cultural construction).

By mapping the cultural ecosocial niches of adults with ADHD, we were able to highlight the multiple and overlapping influences on disorder and coping. By looking beyond intra-individual factors, such as psychological or neurochemical mechanisms, the ecosocial niche theory helped identify how the social world gets ‘under the skin’ and comes to be disorder. This view has consequences for the assessment, formulation, and intervention of those with ADHD. Just as disorder can be shaped by social life, coping can also be sustained by it.

## Footnotes

1 This is the case even when non-referred (random) individuals are sampled, suggesting that this is not merely a case of misdiagnosis (Farrad et al., 2007).

2 There is some evidence that newer ADHD diagnoses in Australian children are statistically more likely to be milder, “leading to diagnostic inflation by widening the definition to include ambiguous or mild symptoms” (Kazda et al., 2021).
